# Incident cytopenia and risk of subsequent myeloid neoplasm in age-related clonal hematopoiesis: a multi-biobank case-control study

**DOI:** 10.1016/j.eclinm.2025.103283

**Published:** 2025-06-04

**Authors:** James Brogan, Ashwin Kishtagari, Robert W. Corty, Yash Pershad, Caitlyn Vlasschaert, Brian Sharber, J. Brett Heimlich, Leo Luo, P. Brent Ferrell, Michael R. Savona, Yaomin Xu, Alexander G. Bick

**Affiliations:** a Department of Medicine, Vanderbilt University Medical Center, Nashville, TN, USA; b Division of Hematology and Oncology, Department of Medicine, Vanderbilt University Medical Center, Nashville, TN, USA; c Division of Rheumatology, Department of Medicine, Vanderbilt University Medical Center, Nashville, TN, USA; d Division of Genetic Medicine, Department of Medicine, Vanderbilt University Medical Center, Nashville, TN, USA; e Department of Medicine, Queen’s University, Kingston, Ontario, Canada; f Division of Cardiovascular Medicine, Department of Medicine, Vanderbilt University Medical Center, Nashville, TN, USA; g Department of Radiation Oncology, Vanderbilt University Medical Center, Nashville, TN, USA; h Department of Bioinformatics, Vanderbilt University Medical Center, Nashville, TN, USA

**Keywords:** CHIP, Cytopenia, CCUS, Myeloid neoplasm

## Abstract

**Background:**

Cross sectional studies have demonstrated patients with clonal hematopoiesis of indeterminate potential (CHIP) are at increased risk of developing multiple adverse outcomes, including cytopenia and myeloid neoplasm (MN). One prior study suggests cytopenia or cytosis is a required intermediate step in disease progression from CHIP to MN.

**Methods:**

We analyzed genomic sequencing data from the NIH All of Us Research Program, Vanderbilt’s BioVU repository, and UK Biobank participants (N = 805,249). The study period ranged from 1 January 2006 to 31 December 2023. Genetic mutations, demographic data, laboratory values, and MN outcomes were used to create a case-control study to estimate the risk of incident cytopenia and MN among cases with CHIP and matched controls without CHIP.

**Findings:**

After applying inclusion and exclusion criteria, the study cohort contained 9374 cases with CHIP and 24,749 matched controls without CHIP. Among the 34,123 participants, 190 (0.56%) developed incident cases of MN and 4151 (12.1%) developed an incident cytopenia. Individuals with CHIP at enrollment who subsequently developed a cytopenia progressed to MN at a rate of 0.5% per year, compared to 0.05% per year for those with CHIP and normal cell counts. Longitudinal analysis across three cohorts demonstrated an increased risk of cytopenia in CHIP patients and identified those at the highest risk of progression. Cytopenia risk factors included smoking (HR = 1.17, 95% CI: [1.05–1.32], P = 5.87 × 10^−3^), male sex (HR = 1.45, 95% CI: [1.30–1.62], P = 2.17 × 10^−11^), variant allele frequency ≥0.20 (HR = 1.36, 95% CI: [1.21–1.54], P = 7.56 × 10^−7^), age ≥65 (HR = 1.41, 95% CI: [1.25–1.57], P = 3.98 × 10^−9^), mean corpuscular volume ≥100 fL (HR = 2.12, 95% CI: [1.68–2.68], P = 2.58 × 10^−10^), red cell distribution width ≥15% (HR = 2.59, 95% CI: [2.26–2.98], P = 1.14 × 10^−40^), mutations in high-risk CHIP genes (HR = 1.48, 95% CI: [1.26–1.75], P = 2.39 × 10^−6^), and ≥2 CHIP mutations (HR = 1.95, 95% CI: [1.61–2.36], P = 9.73 × 10^−12^).

**Interpretation:**

Longitudinal analysis across three large cohorts found that it is rare for patients with CHIP to develop MN without first developing cytopenia. The risk for MN among patients with CHIP resides almost entirely among those with cytopenia. These findings suggest that cytopenia is a critical step in progression from CHIP to MN, underscoring its utility as an endpoint in cancer prevention trials for CHIP patients.

**Funding:**

10.13039/100000002National Institutes of Health, 10.13039/100000861Burroughs Wellcome Fund, 10.13039/100008884Edward P. Evans Foundation, 10.13039/100000875Pew Charitable Trusts, 10.13039/100001353Alexander and Margaret Stewart Trust, Beverly and George Rawlings Directorship.

**Research in context**

**Evidence before this study**

Clonal hematopoiesis of indeterminate potential (CHIP) is associated with an increased risk for myeloid neoplasm (MN), cardiovascular disease, and other aging diseases. When CHIP is present with a concurrent cytopenia, it is termed clonal cytopenia of undetermined significance (CCUS). Prior research has shown that individuals with CCUS are at a much higher risk of progressing to MN compared to those with CHIP alone, leading some to speculate that CCUS is an intermediate state between CHIP and MN. One study demonstrated that nearly all participants with incident MN had a cytopenia or cytosis detected. However, most studies on CHIP and CCUS have relied on cross-sectional data (single time point) rather than following patients longitudinally over time, limiting insights into trajectories of progression from CHIP to CCUS and then potentially to MN. Therefore, key gaps remain in understanding drivers of CHIP progression to best classify which participants with CHIP are most likely to transition to CCUS and subsequent MN.

**Added value of this study**

This study represents the largest longitudinal analysis of CHIP and CCUS, using data across three distinct, diverse population-level biobanks across two continents: the US All of Us Research Program, Vanderbilt BioVU, and the UK Biobank; together encompassing more than 800,000 participants. By incorporating serial blood count measurements over multiple years, this study provides robust quantitative estimates of the incidence of CCUS among participants with CHIP and the rate at which these individuals subsequently progress to MN. We found that the annual rate of progression from CHIP to MN amongst individuals who first developed cytopenia was 0.5% per year, compared to 0.05% per year amongst those who did not develop cytopenia, underscoring cytopenia as a key intermediate step in the pathogenesis of myeloid malignancy. Approximately 13.4% of participants with CHIP developed a cytopenia within 5 years, with gradations of risk by specific genetic and clinical factors.

**Implications of all the available evidence**

This study advances the understanding of CHIP progression by illustrating that cytopenia is a critical intermediate step in the progression of CHIP to MN. Clinically, our data supports most patients with CHIP could be followed with serial blood counts. Our data also highlights cytopenia-free survival as a valuable endpoint for clinical trials aimed at preventing MN among patients with high-risk CHIP. By focusing on cytopenia as an actionable endpoint and further stratifying those most at risk, cancer prevention clinical trials could be designed with reduced sample sizes and shorter follow-up times.

## Introduction

Age-related clonal hematopoiesis (CH) is common among older individuals and is a risk factor for myeloid neoplasms (MN), cardiovascular disease, and multiple other diseases of aging.^1^, ^2^, ^3^, ^4^, ^5^, ^6^, ^7^ CH of indeterminate potential (CHIP) is a type of CH defined by somatic mutations in leukemia driver genes at a variant allele fraction (VAF) ≥ 2% in the absence of cytopenia.^8^ Clonal cytopenia of undetermined significance (CCUS) differs from CHIP in that individuals with CCUS have a persistent unexplained cytopenia in the absence of MN. CHIP and CCUS are observed in 10%–20% of individuals aged over 70 years of age.^1^, ^2^, ^3^^,^^9^ Both CHIP and CCUS are premalignant lesions that confer increased risk of progression to MN. Early risk stratification models predicting progression of premalignant lesions to MN lumped CHIP and CCUS together, with a commonly quoted progression rate of ∼0.5% per year.^10^^,^^11^ However, emerging data suggest that CHIP and CCUS confer distinct risk profiles, as evidenced by the clonal cytopenia risk score, which predicted 2-year cumulative incidence of MN for low (6.4%), intermediate (14.1%) and high-risk (37.2%) groups.^12^ Furthermore, cross-sectional analyses of individuals with CH and evidence of cytopenia based on a single blood draw have found that cytopenia confers increased risk of MN compared to individuals with CHIP who have normal blood counts.^13^, ^14^, ^15^, ^16^ However, these analyses did not employ longitudinal complete blood count data to differentiate between the two disease states over time. One multi-timepoint study including 3359 participants from the Lifelines cohort demonstrated that of 1320 participants with CHIP, 35 developed a MN and only two of these participants did not show cytosis or cytopenia preceding the diagnosis of MN.^17^ Therefore, the rate of progression from CHIP directly to MN may be lower than currently estimated as individuals may progress from CHIP to CCUS before developing MN.

Demonstrating that cytopenia is in the causal pathway of CHIP progression to MN using longitudinal data is important for cancer prevention clinical trial design. Given the very low rate of progression from CHIP to MN, extraordinarily large trial sample sizes would be required to demonstrate clinical benefit of an intervention in patients with CHIP. However, cytopenia is much more common than MN. A cancer prevention trial in patients with CHIP that used cytopenia as a surrogate endpoint would require a smaller sample size and be considerably more feasible to execute. Therefore, it is critically important to rigorously evaluate whether cytopenia is a suitable surrogate endpoint for cancer prevention.

Our ability to predict which individuals with CHIP will develop a persistent cytopenia is limited. Quantifying the incidence of cytopenia in individuals with CHIP and identifying features associated with progression to cytopenia can help improve current risk stratification models and identify individuals with CHIP who may benefit from closer monitoring or intervention.^18^ Here, we analyzed longitudinal complete blood count data from three large diverse biobanks to quantify the incidence of cytopenia among individuals with CHIP at time of enrollment and demonstrate that cytopenia is an intermediate step in the progression of CHIP to MN.

## Methods

### Cohort descriptions

Participant data was obtained from three large observational cohorts: the All of Us Research Program (AoU), UK Biobank (UKB) and Vanderbilt’s BioVU biorepository. The characteristics of eligible study participants included in our study from each cohort are shown in Supplementary Tables S1–S3.

AoU is an ongoing US-based observational cohort study.^19^ AoU whole genome sequencing (WGS) data with median sequencing depth of 40× was available for 245,388 participants who were enrolled from 2017 to 2022.^20^ These participants have linked health outcome data from participant survey questionnaires and electronic health records harmonized to the Observational Medical Outcomes Partnership (OMOP) Common Data Model (CDM) at enrollment and follow-up time points. The median age at enrollment for this cohort is 53 years old (interquartile range, 37 to 65).

BioVU is Vanderbilt’s biorepository of DNA extracted from discarded blood collected during routine clinical testing and linked to de-identified medical records derived from Vanderbilt’s electronic medical record.^21^ WGS data was available for 107,607 adult participants enrolled from 2006 to 2023 with linked electronic health record data harmonized to the OMOP CDM at enrollment and each subsequent healthcare encounter. WGS was performed using Illumina PCR-free whole genome sequencing technology and sequenced on the NovaSeq platform to a median sequencing depth of 35×. The median age at enrollment for this cohort is 50 years old (interquartile range, 34 to 62).

UKB is a UK-based observational cohort study. UKB whole exome sequencing (WES) with median sequencing depth of 40× was available for 454,033 participants aged 40 to 70 at time of DNA collection.^22^ Participants were enrolled from 2007 to 2010 and have questionnaire, physical measurement, laboratory, and medical imaging data available at enrollment and follow-up time points.^23^ Health outcomes since enrollment are tracked from hospitalization general practice health records and death and cancer registries. The median age at enrollment for this cohort is 58 years old (interquartile range, 50 to 63).

AoU and UKB obtained informed consent from all participants as approved by their respective ethics committees. The Vanderbilt University Medical Center’s Institutional Review Board oversees BioVU and approved this study.

### Study design

We conducted a case-control cohort study of participants from AoU, BioVU, and UKB. Individuals were eligible for the study if they had sequencing and multi-timepoint complete blood count (CBC) data, without evidence of cytopenia, acute myeloid leukemia (AML), myelodysplastic syndrome (MDS), or myelofibrosis prior to sequencing. Multi-timepoint CBC was defined as at least three CBC measurements, including one within a year of sequencing and two on or after the date of sequencing. The final CBC measurement had to occur at least 120 days after sequencing or the first CBC measurement, whichever came later (Supplementary Fig. S1). CBC measurements occurring greater than one year before sequencing were not included in the analysis.

Participants with CHIP were matched 1:3 with controls on age, cohort of enrollment, sex, and smoking status. Date of sequencing was not matched between groups. The matching algorithm iterated through each case and identified all controls who met the matching criteria. Identifiers for the potential controls were aggregated into a list and randomly shuffled to prevent selection bias. Up to 3 controls were selected from the randomized pool for each case. If there were only 1 or 2 matched controls, the case was still included in the cohort. If a case had zero matches, they were not included in the cohort. The follow-up period commenced at the date of sequencing and terminated at the earliest occurrence of myelofibrosis, MDS, AML, death, or date of last diagnosis update in each cohort. The date of last diagnosis update was 07/02/2022 in All of Us, 09/01/2023 in BioVU, and 12/31/2020 in UK Biobank at the time of data curation for this study. The primary outcome of interest was incident MN any time after study enrollment.

### CHIP, cytopenia, and MN definitions

Somatic mutations in 58 canonical CHIP driver genes were identified from read-level WGS data in AoU and BioVU, and WES data in UKB, using Genome Analysis Toolkit Mutect2^24^ and ANNOVAR^25^ as previously described.^11^ CH was defined by the presence of somatic mutations in a canonical CHIP driver gene at a VAF ≥2% in AoU, BioVU, and UKB. CH without cytopenia or a diagnosed blood disorder was classified as CHIP. Participants with CHIP who developed an incident cytopenia were not classified as CCUS because bone marrow biopsy reports were not available for all participants. Cytopenias were defined by using a modified version of World Health Organization criteria^8^ (anemia: hemoglobin <12.0 g/dL (females) or 13.0 g/dL (males); thrombocytopenia: platelets <150,000 cells/mL; and leukopenia: white blood cell count <3700 cells/mL). Cytopenias were only deemed to be persistent if there were two consecutive observations of a cytopenia in a single lineage at least 120 days apart without an intervening normal measurement (Supplementary Fig. S1). The date of cytopenia was the first occurrence of the cytopenia that persisted for at least 120 days. MN was defined as AML, MDS, or myelofibrosis. Essential thrombocythemia and polycythemia vera were excluded from the definition of MN in this study because of their phenotypic heterogeneity and the high likelihood of misclassification in electronic health record data.^26^

### Data curation

Date of birth and death, sex, race, laboratory values, self-reported smoking history, and International Classification of Diseases, Ninth and Tenth Revision (ICD-9, ICD-10), codes were extracted across all three biobanks. All laboratory measurement variables were harmonized to a common unit of measure and screened for outlier values. ICD-9 and ICD-10 codes are listed in Supplementary Table S4. There was no missing sequencing or CBC data for the final study cohort as participants had to have undergone sequencing and have multi-timepoint CBC data to be included. Missing ICD codes, self-reported smoking history and date of death for a participant were counted as not occurring. A total of 1188 (3.48%) study participants were missing race data. There were no missing data for date of birth or sex.

### Statistical methods

Statistical analyses were performed using Python (v3.10.12) and survival analyses were performed using R statistical software. Figures were made with matplotlib (v3.7.2) and R (version 4.2.1). Differences in continuous variables between groups were assessed using the Kruskal–Wallis test. When significant, pairwise comparisons were performed using the Mann–Whitney U test, with Bonferroni correction applied to adjust for multiple comparisons. Differences in categorical variables were evaluated using the Chi-square test of independence. All statistical tests were two-sided with statistical significance determined by a P value < 0.05. Cumulative incidence of cytopenia and MN were estimated using Fine–Gray subdistribution hazard modeling within a competing risks framework. The competing risk for incident cytopenia was MN and the competing risk for MN was death. Univariate Cox proportional-hazards models were used to estimate the association between incident cytopenia and somatic mutations in canonical CHIP driver genes at the time of enrollment, comparing participants with CHIP to controls without CHIP, who served as the reference group. These models were controlled for age. Additionally, univariate Cox proportional-hazards models were performed to evaluate the association between incident cytopenia and demographic features (sex, age, ever smoker) as well as laboratory features (MCV and RDW) at the time of enrollment for participants with CHIP. All regression models were adjusted for cohort of enrollment. Sensitivity analyses were performed to assess for differences in length of follow-up between cohorts and subsequent effects on study outcomes. Analyses were repeated on a subgroup of participants across cohorts with at least five years of follow-up. Follow-up duration was defined as the number of days from sequencing until the last registry update for the participant’s cohort or death, whichever occurred first.

### Role of the funding source

The funders of the study had no role in study design, data collection, data management, data analysis, manuscript writing, or the decision to submit the manuscript for publication. JB, AK, RWC, YP, PBF, MRS, and AGB designed the study. Data collection, data processing, statistical analyses, and manuscript writing were all conducted by the investigators.

## Results

**Fig. 1:**
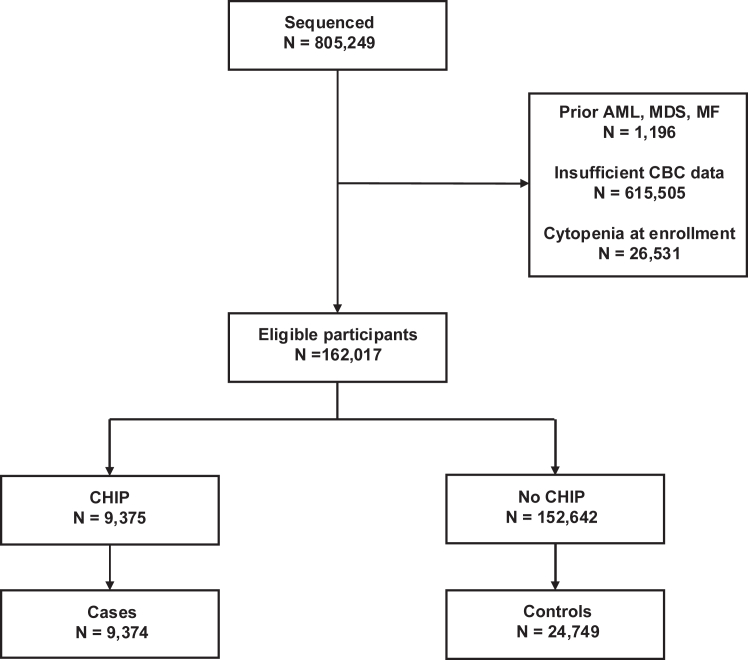
**Flow diagram showing selection of cases and controls.** Abbreviations: AML = acute myeloid leukemia; MDS = myelodysplastic syndrome; MF = myelofibrosis; CBC = complete blood count; CHIP = clonal hematopoiesis of indeterminate potential. Participants were screened from the All of Us Research Program (N = 243,609), Vanderbilt’s BioVU biorepository (N = 107,607), and UK Biobank (N = 454,033). Participants were excluded for prior AML, MDS or MF diagnoses, insufficient CBC data, or cytopenia at enrollment. Cases and controls were matched 1:3 on age ±3 years, gender, and any history of smoking within their respective cohort. The same control was able to be matched to multiple cases.

Sequencing data were available for 805,249 participants, with 162,017 participants meeting inclusion criteria, including 9375 with CHIP (Fig. 1). Of those excluded, 615,505 participants lacked sufficient serial CBC data, resulting in a final matched case-control cohort of 9374 cases and 24,749 controls (Fig. 1, Supplementary Fig. S2). Characteristics of cases and controls are detailed in Table 1. The age distribution was significantly different between cases and controls (P < 1 × 10^−6^). There was no difference in sex (P = 0.88) or smoking history (P = 0.67) between cases and controls. There were inter-cohort differences (Supplementary Tables S1–S3). The median age of cases in UKB was 62.4 years (interquartile range, 57.5–66.2) which was significantly younger than the median age of 69.2 years (interquartile range, 60.9–75.6) in AoU and 65.2 years (interquartile range, 55.6–73.0) in BioVU. The year of sample collection for sequencing impacted the length of follow-up for each cohort. All of Us participants were enrolled from 2017 to 2022 with a median [IQR] follow-up time of 3.1 years [2.7, 3.6] for cases and 3.1 years [2.6, 3.6] for controls. UK Biobank participants were enrolled from 2007 to 2010, and their median follow-up time was 11.5 years [11.0, 12.1] for cases and 11.6 years [11.1, 12.1] for controls. In comparison, BioVU participants were enrolled continuously from 2006 to 2023, and their median follow-up time was 10.2 years [6.7, 13.6] for cases and 10.2 years [6.5, 13.6] for controls. There was a statistically significant difference in median follow-up time across the cohorts (P < 1 × 10^−6^). All pairwise comparisons were also statistically significant (P < 1 × 10^−6^).

**Table 1:** Characteristics of cases and controls.^a^

Characteristic	Cases (N = 9374)	Controls (N = 24,749)
Age—median [IQR], year	63.6 [57.7, 68.4]	63.1 [57.1, 67.7]
Female—no. (%)	5237 (55.9)	13,898 (56.2)
Any smoking history—no. (%)	4812 (51.3)	12,639 (51.1)
Cohort		
All of Us Research Program—no. (%)	1937 (20.7)	5109 (20.7)
Vanderbilt BioVU biorepository—no. (%)	1622 (17.3)	4314 (17.4)
UK Biobank—no. (%)	5815 (62.0)	15,326 (61.9)
Laboratory values^b^		
Hemoglobin—median [IQR], g/dL	14.0 [13.2, 14.9]	14.0 [13.2, 14.9]
Platelet count—median [IQR], (10^9^ cells/L)	247 [210, 290]	244 [208, 286]
White blood cells—median [IQR], (10^9^ cells/L)	6.9 [5.7, 8.3]	6.7 [5.6, 8.1]
Mean corpuscular volume—median [IQR], fL	91.0 [88.1, 94.0]	91.0 [88.1, 94.0]
Red cell distribution width—median [IQR], %	13.5 [13.0, 14.1]	13.4 [12.9, 14.0]
Follow-up—median [IQR], year^c^	11.2 [4.8, 12.0]	11.2 [4.8, 12.0]
Type of incident cytopenia^d^		
Anemia—no. (%)	1013 (10.8)	2299 (9.3)
Thrombocytopenia—no. (%)	260 (2.8)	587 (2.4)
Leukopenia—no. (%)	136 (1.5)	271 (1.1)
Incident cytopenia—no. (%)	1260 (13.4)	2882 (11.6)
Incidence of cytopenia—per 1000 person-years	29	25
Time to cytopenia—median [IQR], years^e^	2.3 [0.7, 4.2]	1.9 [0.5, 3.0]
Incident AML, MDS, MF—no. (%)	98 (1.1)	92 (0.4)
AML—no. (%)	39 (0.4)	39 (0.2)
MDS—no. (%)	47 (0.5)	52 (0.2)
MF—no. (%)	12 (0.1)	1 (0.004)
Time from cytopenia to MN^f^—median [IQR], years^g^	1.5 [0.6, 3.2]	2.6 [0.7, 5.5]
Death—no. (%)	1045 (11.2)	2204 (8.9)

IQR: interquartile range; AML: acute myeloid leukemia; MDS: myelodysplastic syndrome; MF: myelofibrosis; MN: myeloid neoplasm.

a Cases and controls were matched 3:1 on age ±3 years, sex, any smoking history. These characteristics were checked for balance after matching. The age distributions were significantly different (P < 1 × 10^−6^) while the sex (0.88) and smoking history (0.67) were not significantly different.

b Hematologic measurements were obtained from complete blood count obtained nearest to time of sequencing.

c Follow-up time is the number of years from sequencing to death or last follow-up in each cohort, whichever is earliest. All of Us cutoff date 07/02/2022, BioVU cutoff date 09/01/2023 and UK Biobank cutoff date 12/31/2020.

d Cytopenia definitions: anemia (hemoglobin < 12.0 g/dL for females or 13.0 g/dL for males), thrombocytopenia (platelet count < 150,000 cells/mL), leukopenia (white blood cell count < 3700 cells/mL). Cytopenias were only counted if there were two consecutive observations of a cytopenia in a single lineage at least 120 days apart without an intervening normal measurement.

e Times only reported for participants who developed cytopenia without subsequent myeloid neoplasm or cytopenia before myeloid neoplasm.

f Myeloid neoplasms are defined as acute myeloid leukemia, myelodysplastic syndrome, myelofibrosis.

g Times reported for participants who developed cytopenia prior to myeloid neoplasm.

**Fig. 2:**
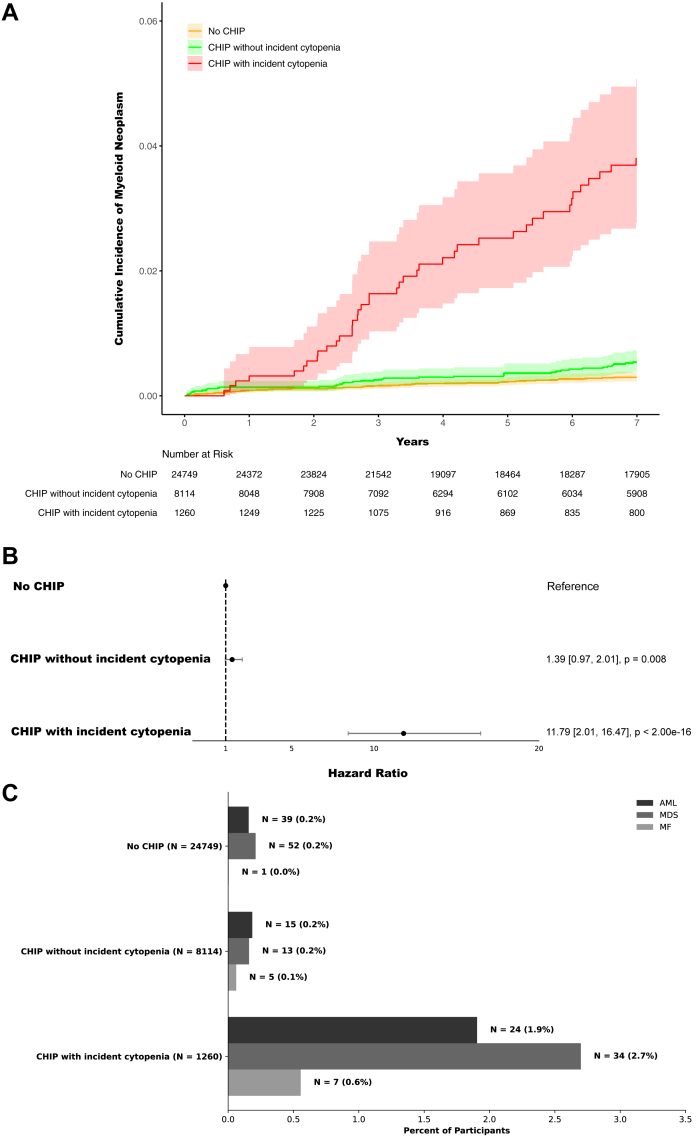
**Risk of incident AML, MDS, MF stratified by CHIP status at enrollment and incident cytopenia.** Abbreviations: AML = acute myeloid leukemia; MDS = myelodysplastic syndrome; MF = myelofibrosis; MN = myeloid neoplasm; CHIP = clonal hematopoiesis of indeterminate potential. (A) Risk of incident AML, MDS, MF in participants with CHIP stratified by incident cytopenia status compared to matched controls across All of Us Research Program, Vanderbilt’s BioVU biorepository, and UK Biobank. (B) Hazard ratios for incident AML, MDS, MF were calculated for cases and controls stratified by incident cytopenia using Cox proportional-hazards models adjusted for cohort, age, sex, gender, and smoking history. (C) Subtypes of MN among participants with CHIP stratified by incident cytopenia status and matched controls. Hazard ratios were calculated in a model with participants without CHIP as the reference population. Forest plot indicates HR and 95% confidence intervals.

First, we assessed the rate of MN in individuals with CHIP and matched controls stratifying based on incident cytopenia status (Fig. 2). Amongst the 34,123 participants in the matched case-control set, 190 (0.56%) developed incident cases of MN and 4151 (12.1%) developed incident cytopenia. The most common first MN was myelodysplastic syndrome with 99 cases, followed by 78 cases of acute myeloid leukemia and 13 cases of myelofibrosis. Participants with CHIP who developed incident cytopenia had significantly higher risk of subsequent MN compared to those without cytopenia (HR = 8.5, 95% confidence interval: [2.7–26.3], P = 2.0 × 10^−16^). Participants with CHIP who did not develop an incident cytopenia had a rate of subsequent MN that was nearly the same as the population controls. Among 1260 individuals with CHIP who developed an incident cytopenia, 65 (5.2%) developed a MN during follow-up with a median time to MN of 4.4 years (interquartile range, 2.6 to 7.4) and an overall MN progression rate of 0.5% per year. Among 8114 individuals with CHIP who did not develop cytopenia, 33 (0.4%) developed a MN during follow-up with a median time to diagnosis of 5.3 years (interquartile range, 3.0 to 6.7) and an overall MN progression rate of 0.06% per year. Among 24,749 individuals without CHIP, 92 (0.4%) developed a MN during follow-up with a median time to diagnosis of 3.9 years (interquartile range, 1.2 to 7.3) and an overall MN progression rate of 0.04% per year. The time from cytopenia onset to MN progression was significantly shorter in individuals with CHIP mutations (median 1.4 years, interquartile range, 0.5–3.4) than in individuals without CHIP mutations (median 2.6 years, interquartile range, 0.7–5.5). In total, this data highlights that individuals with CHIP who develop cytopenia are at risk of progressing to MN, while individuals with CHIP who do not develop cytopenia are not at any meaningfully greater risk of MN progression than the general population who does not have CHIP.

Next, we quantified the incidence of cytopenia in individuals with CHIP and matched controls. Median time at risk was 5.08 years in cases (interquartile range, 2.51 to 6.50) and 5.16 years in controls (interquartile range 2.53–6.54). Incident cytopenia occurred in 1260 (13.4%) cases and 2882 (11.64%) controls, with anemia being the major cause of cytopenia. Fine–Gray modeling for incident cytopenia with competing risk of MN demonstrated that individuals with CHIP had a significantly increased risk of incident cytopenia compared to matched controls (HR = 1.17, 95% confidence interval: [1.10–1.25], P = 2.5 × 10^−6^) with consistency across all three cohorts (Supplementary Fig. S3). This consistency of effect was particularly notable given the cumulative incidence of cytopenia over two years varied considerably across the cohorts, with 15.9% of cases in BioVU and 13.1% in AoU compared to just 2.9% of cases in UKB.

**Fig. 3:**
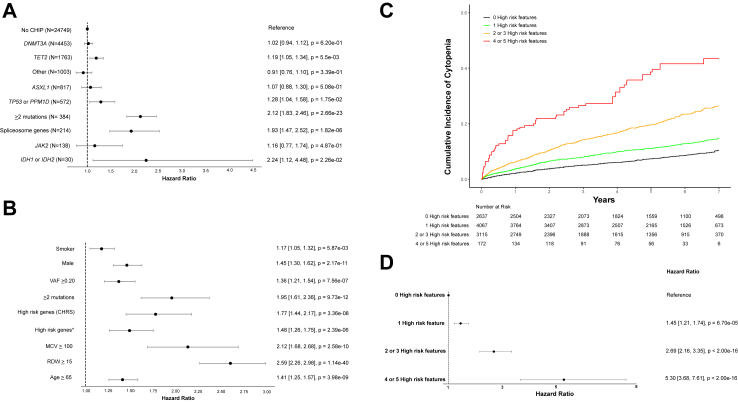
**Features influencing risk of cytopenia in participants with CHIP.** Abbreviations: VAF = variant allele fraction; CHRS = clonal hematopoiesis risk score; MCV = mean corpuscular volume (femtoliters); RDW = red cell distribution width (%); CHIP = clonal hematopoiesis of indeterminate potential. (A) Univariate Cox regression analyses for incident cytopenia by specific CHIP genotypes at the time of enrollment for participants with CHIP and controls without CHIP serving as the reference group. Analyses were adjusted for age and cohort of enrollment. (B) Univariate Cox regression analyses for incident cytopenia by baseline characteristic at the time of enrollment for participants with CHIP with adjustment for cohort of enrollment. The variable high-risk genes (CHRS) indicates a participant had at least one mutation in the following genes: *SRSF2*, *SF3B1*, *ZRSR2*, *IDH1*, *IDH2*, *FLT3*, *RUNX1*, or *JAK2*. The variable high-risk genes∗ indicates a participant had at least one mutation in the following genes: *TP53*, *PPM1D*, *SF3B1*, *SRSF2*, *U2AF1*, *ZRSR2*, *IDH1*, or *IDH2*. (C) Cumulative incidence curve for cytopenia in participants with CHIP across All of Us Research Program, Vanderbilt’s BioVU biorepository, and UK Biobank stratified by the number of high-risk features they had at time of enrollment. High risk features were defined as age ≥65 years, male gender, ≥2 CHIP mutations, MCV ≥100 fL, RDW≥15%, or the presence of at least one high-risk CHIP mutation (*SRSF2*, *SF3B1*, *ZRSR2*, *IDH1*, *IDH2*, *FLT3*, *RUNX1*, or *JAK2*). (D) Hazard ratios for incident cytopenia were calculated for high-risk feature strata using Cox proportional hazards models adjusted for cohort, age, sex, gender, and smoking history. Hazard ratios were calculated in a model with zero high-risk features as the reference population. Forest plot indicates HR and 95% confidence intervals.

We then identified high-risk genetic features that amplify cytopenia risk in participants with CHIP. Cox regression analyses controlling for cohort of enrollment were conducted to identify which CHIP driver mutations conferred the highest risk of incident cytopenia. CHIP driven by a mutation in *TET2* was found to confer an increased risk of incident cytopenia compared to individuals without CHIP (HR = 1.19, 95% confidence interval: [1.05–1.34], P = 5.6 × 10^−3^), while CHIP driven by *DNMT3A, ASXL1,* and *JAK2* did not (Fig. 3A). Aggregating CHIP driver mutations by mechanism demonstrated increased risk of incident cytopenia among participants with driver mutations in the *TP53* pathway (*TP53* or *PPM1D*; HR = 1.28, 95% confidence interval: [1.04–1.58], P = 1.8 × 10^−2^), in spliceosome genes (*SF3B1*, *SRSF2*, *U2AF1*, or *ZRSR2*; HR = 1.93, 95% confidence interval: [1.47–2.52], P = 1.8 × 10^−6^), and CHIP driven by a mutation in *IDH1* or *IDH2* which are commonly mutated in MN (HR = 2.24, 95% confidence interval: [1.12–4.48], P = 2.3 × 10^−2^). Individuals with two or more CHIP mutations were also at significantly increased risk for incident cytopenia (HR = 2.12, 95% confidence interval: [1.83–2.46], P = 2.7 × 10^−23^). All effects were directionally consistent across cohorts (Supplementary Fig. S4).

Clinical factors that increase risk for incident cytopenia were assessed using univariate Cox regression analysis controlling for cohort of enrollment. Smoking status, male sex, VAF ≥0.20, ≥2 CHIP mutations, mean corpuscular volume (MCV) ≥ 100 fL, red cell distribution width (RDW) ≥ 15%, and age ≥65 years all demonstrated statistical significance (Fig. 3B). We also compared a previously nominated set of high-risk CHIP mutations^15^^,^^27^ (*SRSF2*, *SF3B1*, *ZRSR2*, *IDH1*, *IDH2*, *FLT3*, *RUNX1*, *JAK2*) to those that were significant in our analysis (*SF3B1*, *SRSF2*, *U2AF1*, ZRSR2, *IDH1*, *IDH2*, *TET2*, *TP53*, *PPM1D*). These factors were directionally consistent across all three cohorts (Supplementary Fig. S5). In AoU, male sex, MCV ≥100 fL, and RDW ≥15% conferred increased risk of cytopenia. In BioVU and UKB, all identified risk factors for incident cytopenia demonstrated significance except for VAF in BioVU and smoking status in UKB.

Individuals with multiple high-risk features (age ≥65 years, male gender, ≥2 CHIP mutations, MCV ≥100 fL, RDW ≥15%, or the presence of any high-risk CHIP mutations) had a significantly greater risk of incident cytopenia (Fig. 3C and D). This increase in risk of incident cytopenia among participants with multiple high-risk features was consistent across cohorts (Supplementary Figs. S6 and S7). For example, BioVU participants with CHIP and no high-risk features had an 8.1% incidence of cytopenia at 2 years (95% confidence interval, 5.6%–11.8%). This increased to 21.0% among participants with 2 or 3 risk factors (95% confidence interval, 18.0%–24.4%) and 45.1% among participants with 4 or 5 risk factors (95% confidence interval, 33.3%–58.9%).

We performed sensitivity analyses to assess the robustness of our findings given differences in date of sequencing and resultant length of follow-up between cohorts (Supplementary Fig. S8). Analyses were repeated on a subgroup of participants across cohorts who had at least five years of follow-up. Cytopenia risk factors including smoking, male sex, VAF ≥ 0.20, age ≥ 65 years, MCV ≥ 100 fL, RDW ≥ 15%, high-risk CHIP mutations, and ≥2 CHIP mutations continued to confer increased risk of incident cytopenia (Supplementary Fig. S9). Small decreases in the hazard ratios for individual risk factors were observed following the exclusion of participants without at least 5 years of follow-up or those who died during the study period. These higher-risk individuals were more prevalent in the AoU and BioVU cohorts.

In this subgroup, genetic features including *TP53* or *PPM1D* mutations and *IDH1* or *IDH2* mutations did not confer a significant increase in risk of incident cytopenia compared to controls without CHIP in this analysis (Supplementary Fig. S10). The remainder of the genetic features remained directionally consistent with the results as displayed in Fig. 3A. Individuals with multiple high-risk features, as defined above, had a significantly greater risk of incident cytopenia (Supplementary Fig. S11) consistent with the main analysis. Furthermore, individuals in the subgroup with CHIP who went on to develop a cytopenia were at increased risk of progressing to MN, while those with CHIP who did not develop cytopenia were not at any meaningfully greater risk of MN progression than the general population without CHIP (Supplementary Fig. S12).

## Discussion

Longitudinal analysis of 34,123 individuals, including 9374 individuals with CHIP, demonstrate that cytopenia is a critical intermediate step in the progression from CHIP to MN. This observation is implicit in the conceptual framework of progression from CHIP to CCUS to MN but has not previously been quantified from longitudinal data at scale.^17^ These findings should be reassuring to both patients and clinicians caring for patients with CHIP as it highlights cytopenia as a biomarker in the causal pathway to malignancy. Our observations permit several conclusions.

First, we found the absolute risk of developing a MN among participants with CHIP in the absence of cytopenia to be 0.05%–0.1% per year, with the upper bound being ten-fold lower than the 1%/year annual risk previously reported.^1^^,^^11^ Participants with CHIP who did not develop an incident cytopenia have nearly identical risk as individuals without CHIP. This highlights the low-risk nature of CHIP and suggests that changes in markers of hematopoiesis, such as MCV and RDW, in this population could be monitored as a harbinger for future cytopenia and subsequent MN. We did identify a very small population of patients who develop cytopenia without CHIP and subsequently developed MN. We posit that this subset could be driven by somatic copy number alterations or small CHIP clones which were undetected at the time of sequencing. Individuals with detectable CHIP mutations who went on to develop cytopenia and MN had a median latency period of approximately 5 years, consistent with latency periods before AML diagnosis in prior studies.^5^^,^^28^ This latency period provides an opportunity for early intervention with potential disease-modifying therapies before malignant transformation occurs.

Second, the existence of high-risk subsets of CHIP that can be identified based on a combination of routine clinical and genomic features enables the clinical development of blood cancer prevention therapies. For example, a hypothetical intervention that reduces the risk of individuals with CHIP from developing a cytopenia by a factor of 2 in a population similar to BioVU, would require 868 patient-years of follow up if all CHIP participants are enrolled. However, if only enrolled CHIP participants with 4 or more risk factors (Fig. 3) are enrolled, only 174 patient-years of follow up would be required.

Third, patients with CHIP have a substantial annual risk of developing a cytopenia, ranging from 1 to 15% in the UK Biobank to 10–35% in the US-based All of Us and BioVU cohorts with degree of risk depending on the number of high-risk features. This increased risk of cytopenia among patients with CHIP has long been presumed, implicit in the conceptual framework of progression from CHIP to CCUS to MN but has not previously been quantified. The relative risk of cytopenia was remarkably consistent across all three cohorts, supporting the interpretation that the observed signal reflects an underlying biological process rather than a cohort-specific artifact. Individuals with *TP53* or *PPM1D* mutations demonstrated a 28% increased risk of developing an incident cytopenia, which may be attributable to prior cytotoxic chemotherapy exposure, a factor not controlled for in this analysis. In the sensitivity analysis, subgroups with *TP53* or *PPM1D* mutations and those with *IDH1* or *IDH2* mutations did not demonstrate a statistically significant association with cytopenia risk, diverging from the findings in the primary analysis. We suggest that this discrepancy may be due to the exclusion of the highest risk participants, those who either lacked 5 years of follow-up or died from causes related to their high-risk genetic features. Given the substantial risk of cytopenia, patients with multiple high-risk features may benefit from regular monitoring for cytopenia progression. Modeling the optimal timing for CBC monitoring is an important future direction.

Lastly, routine laboratory measurements including MCV and RDW have been previously identified as prognosticating risk of progression from clonal hematopoiesis to MN are important risk factors for predicting an incident cytopenia in individuals with CHIP.^15^^,^^27^^,^^28^ Although this concordance may be explained in part by the use of UK Biobank as one of the three studies in our analyses and in several prior studies, the strong consistency of results across all three cohorts, suggests that the results generalize from healthy individuals recruited in the UK to the US population (All of Us) and individuals identified in health care settings (BioVU). Furthermore, our analysis builds on *Abelson* et al. who found significantly elevated RDW measurements several years before AML diagnosis relative to age and sex-matched controls in an independent cohort.^28^ Their results suggests that evolving *de novo* AML may sometimes have a prodrome with subtle but discernable clinical manifestations which we corroborated and extended by demonstrating that elevated RDW is a risk factor for incident cytopenia on the pathway from CHIP to MN.

Our study has several limitations. First, while developing an incident cytopenia serves as a strong biomarker of high-risk CH, some individuals may have had undetected CHIP clones at enrollment. We derived CHIP calls based on both WES and WGS, this approach carries a lower depth of coverage compared to targeted sequencing approaches.^29^ Our analysis reduced sensitivity for detecting CHIP clones that make up only a small fraction of the blood. Targeted myeloid next generation sequencing panels may identify individuals at risk for MN with higher sensitivity, better classifying participants into their appropriate risk groups for cancer prevention clinical trials. Extending our analyses to small CHIP clones is an important area of future study. Second, there were notable differences in the rate of cytopenia across the cohorts, likely due to multiple factors. UK Biobank participants have been shown to be substantially healthier than the UK population in general,^30^ while participants recruited from a healthcare system settings such as those recruited in All of Us and BioVU were substantially less healthy than the US population in general.^31^ UK Biobank participants were younger than those in the All of Us and BioVU cohorts. The number of CBCs per participant also differed by cohort, which is perhaps due to different practice patterns. Third, we did not have bone marrow biopsy data for each participant and had to classify participants as having CHIP with incident cytopenia rather than CCUS because the participant could have already developed MDS. Fourth, our study did not include somatic copy number alterations, which are known to accumulate with age and are associated with subsequent development of blood count abnormalities and mortality from hematologic malignancy.^32^ Future risk models could be improved by incorporating these genetic factors. Addressing these limitations in future research will be crucial for a more comprehensive understanding of the relationship between CHIP, cytopenia and MN. Lastly, our study was designed to better understand the relationship between CHIP, incident cytopenia development and progression to MN. While cytopenias are expected clinical manifestations of MDS and AML, the observed association between higher-risk CHIP mutations (particularly *TET2*) and incident cytopenia aligns with this pathogenic trajectory. However, the lack of association between *JAK2* mutations and cytopenia development merits consideration of alternative phenotypic changes preceding myeloproliferative neoplasms (MPNs). Specifically, proliferative phenotypes not meeting formal MPN criteria are frequently associated with *JAK2* mutations and may represent a distinct pathway to malignant transformation. In conclusion, our study provides pivotal insights into trajectories from CHIP to more advanced disease stages, highlighting the intermediate step of cytopenia as a crucial marker in the trajectory from CH to MN. We propose that cytopenia-free survival represents a key endpoint for future cancer prevention clinical trials for patients with CHIP given that our data shows that cytopenia is a required step for malignancy progression. Our ability to identify individuals at the highest risk of developing cytopenia and subsequent MN will enable the efficient design and execution of interventional trials targeting this population.

## Contributors

JB, AK, RWC, YP, PBF, MRS and AGB conceived and designed the study. JB, AK, RWC, YP, and AGB had full access to and verified the study data. JB performed primary analysis and AK prepared figures and tables. JB, AK, RWC, YP and AGB contributed to interpretation of the data. BH, LL, PBF and MRS assisted with refinement of the study design. YX and AGB contributed to data acquisition. RWC, YP, CV and BS performed somatic mutation calls in the All of Us Research Program, UK Biobank, and Vanderbilt’s BioVU biorepository. JB, AK and AGB wrote the first draft of the manuscript which was critically revised for important intellectual content with feedback from all authors.

## Data sharing statement

Data for UK Biobank and All of Us can be made available to researchers by registering with each respective biobank and obtaining clearance per their processes. Please email the corresponding author for further information to access Vanderbilt’s BioVU and Synthetic Derivative databases.

## Declaration of interests

All authors have completed the International Committee of Medical Journal Editors disclosure of interest form and declare the following disclosures. AGB is on the scientific advisory board and has received personal fees from TenSixteen Bio unrelated to the present work. MRS receives equity from Empath Biosciences, Karyopharm, Ryvu; consulting fees from BMS, Geron, Karyopharm, Rigel, Ryvu, Taiho and Treadwell; and research fees to the institution from Incyte, Prelude, Takeda, Taiho. PBF receives research fees to the institution from Novartis.
