# Supplementary material for: Incident cytopenia and risk of subsequent myeloid neoplasm in age-related clonal hematopoiesis: a multi-biobank case-control study
Source: eClinicalMedicine. 2025 Jun 4;84:103283. doi: 10.1016/j.eclinm.2025.103283 (PMC12169786; doi:10.1016/j.eclinm.2025.103283)
Supplement: Supplementary Figs. S1–S12 and Tables S1–S4 [file mmc1.docx]

**Supplementary Appendix**

**Incident cytopenia and risk of subsequent myeloid neoplasm in age-related clonal hematopoiesis: a multi-biobank case-control study**

James Brogan,^1^ Ashwin Kishtagari,^2^ Robert W. Corty,^3^ Yash Pershad,^4^ Caitlyn Vlasschaert,^5^ Brian Sharber,^4^ J. Brett Heimlich,^6^ Leo Luo,^7^ P. Brent Ferrell,^2^ Michael R. Savona,^2^ Yaomin Xu,^8^ and Alexander G. Bick^4^

^1^Department of Medicine, Vanderbilt University Medical Center, Nashville, TN, USA; ^2^Department of Medicine, Division of Hematology and Oncology, Vanderbilt University Medical Center, Nashville, TN, USA; ^3^Department of Medicine, Division of Rheumatology, Vanderbilt University Medical Center, Nashville, TN, USA; ^4^Department of Medicine, Division of Genetic Medicine, Vanderbilt University Medical Center, Nashville, TN, USA; ^5^Department of Medicine, Queen’s University, Kingston, Ontario, Canada; ^6^Department of Medicine, Division of Cardiovascular Medicine, Vanderbilt University Medical Center, Nashville, TN, USA; ^7^Department of Radiation Oncology, Vanderbilt University Medical Center, Nashville, TN, USA; and ^8^Department of Bioinformatics, Vanderbilt University Medical Center, Nashville, TN, USA

JB and AK contributed equally to this study.

**Table of Contents**

1. **Supplemental Tables**

Table S1 – Characteristics of cases and controls in the All of Us Research Program

Table S2 – Characteristics of cases and controls in Vanderbilt’s BioVU biorepository

Table S3 – Characteristics of cases and controls in UK Biobank

Table S4 – ICD-9 and ICD 10 codes used to define myeloid neoplasms

1. **Supplemental Figures**

Figure S1 – Multi-timepoint complete blood count eligibility and cytopenia criteria

Figure S2 – Flow diagram showing selection of cases and controls across cohorts

Figure S3 – Risk of incident cytopenia in participants with CHIP

Figure S4 – Risk of incident cytopenia by genotype across cohorts

Figure S5 – Risk of incident cytopenia by baseline characteristic across cohorts

Figure S6 – Risk of incident cytopenia stratified by participant risk factor profiles across cohorts

Figure S7 – Cumulative incidence of cytopenia two years after enrollment in participants with CHIP in each

biobank stratified by number of high-risk features

Figure S8 – Participant year of sequencing by cohort of enrollment

Figure S9 – Risk of incident cytopenia by baseline characteristic in subgroup with at least 5 years of follow-up

Figure S10 – Risk of incident cytopenia by genotype in subgroup with at least 5 years of follow-up

Figure S11 – Risk of incident cytopenia stratified by participant risk factor profiles in subgroup with at least 5 years of

follow-up

Figure S12 – Risk of incident AML, MDS, MF stratified by CHIP status at enrollment, incident cytopenia

**Table S1. Characteristics of cases and controls in the All of Us Research Program.^*^**

| Characteristic | **Cases**  **(N=1,937)** | **Controls**  **(N=5,109)** |
| --- | --- | --- |
| Age – median [IQR], year | 69.2 [60.9, 75.6] | 68.2 [59.8, 74.7] |
| Female – no. (%) | 1,124 (58.0) | 2,983 (58.4) |
| Any smoking history – no. (%) | 639 (33.0) | 1,710 (33.5) |
| Laboratory values^†^ |  |  |
| Hemoglobin – median [IQR], g/dL | 13.7 [13.0, 14.6] | 13.8 [12.9, 14.6] |
| Platelet count – median [IQR], (10^9^ cells/L) | 236 [199, 281] | 233 [198, 275] |
| White blood cells – median [IQR], (10^9^ cells/L) | 6.8 [5.7, 8.6] | 6.7 [5.5, 8.4] |
| Mean corpuscular volume – median [IQR], fL | 90.8 [87.7, 94.0] | 90.8 [87.6, 94.0] |
| Red cell distribution width – median [IQR], % | 13.6 [13.0, 14.4] | 13.5 [12.9, 14.2] |
| Follow-up – median [IQR], year^‡^ | 3.1 [2.7, 3.6] | 3.1 [2.6, 3.6] |
| Type of incident cytopenia^§^ |  |  |
| Anemia – no. (%) | 237 (12.2) | 499 (9.8) |
| Thrombocytopenia – no. (%) | 52 (2.7) | 108 (2.1) |
| Leukopenia – no. (%) | 24 (1.24) | 61 (1.2) |
| Incident cytopenia – no. (%) | 284 (14.7) | 613 (12.0) |
| Incidence of cytopenia – per 1,000 person-years | 67 | 56 |
| Death – no. (%) | 37 (1.9) | 104 (2.0) |

IQR: interquartile range.

^*^ Cases and controls were matched 3:1 on age ± 3 years, sex, any smoking history.

^†^ Hematologic measurements were obtained from complete blood count obtained nearest to time of sequencing.

^‡^ Follow-up time is the number of years from sequencing to death or last follow-up in each cohort, whichever is earliest. All of Us cutoff date 07/02/2022.

^§^ Cytopenia definitions: anemia (hemoglobin < 12.0 g/dL for females or 13.0 g/dL for males), thrombocytopenia (platelet count < 150,000 cells/μL), leukopenia (white blood cell count < 3,700 cells/μL). Cytopenias were only counted if there were two consecutive observations of a cytopenia in a single lineage at least 120 days apart without an intervening normal measurement.

**Table S2. Characteristics of cases and controls in Vanderbilt’s BioVU biorepository.^*^**

| Characteristic | **Cases**  **(N=1,622)** | **Controls**  **(N=4,314)** |
| --- | --- | --- |
| Age – median [IQR], year | 65.2 [55.6, 73.0] | 63.8 [54.6, 72.0] |
| Female – no. (%) | 915 (56.4) | 2,437 (56.5) |
| Any smoking history – no. (%) | 456 (28.1) | 1,209 (28.0) |
| Laboratory values^†^ |  |  |
| Hemoglobin – median [IQR], g/dL | 13.8 [13.0, 14.7] | 13.8 [12.9, 14.8] |
| Platelet count – median [IQR], (10^9^ cells/L) | 246 [202, 298] | 241 [202, 288] |
| White blood cells – median [IQR], (10^9^ cells/L) | 7.2 [5.9, 9.1] | 7.1 [5.8, 8.8] |
| Mean corpuscular volume – median [IQR], fL | 91.0 [88.0, 94.0] | 91.0 [88.0, 94.0] |
| Red cell distribution width – median [IQR], % | 13.6 [13.0, 14.4] | 13.4 [12.9, 14.2] |
| Follow-up – median [IQR], year^‡^ | 10.2 [6.7, 13.6] | 10.2 [6.5, 13.6] |
| Type of incident cytopenia^§^ |  |  |
| Anemia – no. (%) | 376 (23.2) | 883 (20.5) |
| Thrombocytopenia – no. (%) | 84 (5.2) | 205 (4.8) |
| Leukopenia – no. (%) | 41 (2.5) | 81 (1.9) |
| Incident cytopenia – no. (%) | 432 (26.6) | 1,014 (23.5) |
| Incidence of cytopenia – per 1,000 person-years | 58 | 48 |
| Death – no. (%) | 185 (11.4) | 407 (9.4) |

IQR: interquartile range.

^*^ Cases and controls were matched 3:1 on age ± 3 years, sex, any smoking history.

^†^ Hematologic measurements were obtained from complete blood count obtained nearest to time of sequencing.

^‡^ Follow-up time is the number of years from sequencing to death or last follow-up in each cohort, whichever is earliest. BioVU cutoff date 09/01/2023.

^§^ Cytopenia definitions: anemia (hemoglobin < 12.0 g/dL for females or 13.0 g/dL for males), thrombocytopenia (platelet count < 150,000 cells/μL), leukopenia (white blood cell count < 3,700 cells/μL). Cytopenias were only counted if there were two consecutive observations of a cytopenia in a single lineage at least 120 days apart without an intervening normal measurement.

**Table S3. Characteristics of cases and controls in UK Biobank.^*^**

| Characteristic | **Cases**  **(N=5,815)** | **Controls**  **(N=15,326)** |
| --- | --- | --- |
| Age – median [IQR], year | 62.4 [57.5, 66.2] | 62.3 [57.0, 65.9] |
| Female – no. (%) | 3,198 (55.0) | 8,478 (55.3) |
| Any smoking history – no. (%) | 3,717 (63.9) | 9,720 (63.4) |
| Laboratory values^†^ |  |  |
| Hemoglobin – median [IQR], g/dL | 14.1 [13.4, 15.0] | 14.10 [13.3, 14.9] |
| Platelet count – median [IQR], (10^9^ cells/L) | 250 [216, 290] | 249 [214, 289] |
| White blood cells – median [IQR], (10^9^ cells/L) | 6.8 [5.7, 8.0] | 6.6 [5.6, 7.8] |
| Mean corpuscular volume – median [IQR], fL | 91.1 [88.5, 93.8] | 91.1 [88.6, 93.8] |
| Red cell distribution width – median [IQR], % | 13.4 [13.0, 13.9] | 13.4 [12.9, 13.9] |
| Follow-up – median [IQR], year^‡^ | 11.5 [11.0, 12.1] | 11.6 [11.1, 12.1] |
| Type of incident cytopenia^§^ |  |  |
| Anemia – no. (%) | 400 (6.9) | 917 (6.0) |
| Thrombocytopenia – no. (%) | 124 (2.1) | 274 (1.8) |
| Leukopenia – no. (%) | 71 (1.2) | 129 (0.8) |
| Incident cytopenia – no. (%) | 553 (9.5) | 1,255 (8.2) |
| Incidence of cytopenia – per 1,000 person-years | 17 | 15 |
| Death – no. (%) | 823 (14.2) | 1,693 (11.1) |

IQR: interquartile range.

^*^ Cases and controls were matched 3:1 on age ± 3 years, sex, any smoking history.

^†^ Hematologic measurements were obtained from complete blood count obtained nearest to time of sequencing.

^‡^ Follow-up time is the number of years from sequencing to death or last follow-up in each cohort, whichever is earliest. UK Biobank cutoff date 12/31/2020.

^§^ Cytopenia definitions: anemia (hemoglobin < 12.0 g/dL for females or 13.0 g/dL for males), thrombocytopenia (platelet count < 150,000 cells/μL), leukopenia (white blood cell count < 3,700 cells/μL). Cytopenias were only counted if there were two consecutive observations of a cytopenia in a single lineage at least 120 days apart without an intervening normal measurement.

**Table S4. ICD-9 and ICD-10 codes used to define myeloid neoplasms**

| **Disease** | **ICD-9** | **ICD-10** |
| --- | --- | --- |
| Acute myeloid leukemia | 205.0, 205.00, 205.01, 205.02 | C92.0, C92.00, C92.01, C92.02, C92.4, C92.40, C92.41, C92.42, C92.5, C92.50, C92.51, C92.52, C92.6, C92.60, C92.61, C92.62, C92.A, C92.A0, C92.A1, C92.A2 |
| Essential thrombocythemia | 238.71 | D47.3 |
| Myelodysplastic syndrome | 238.72, 238.73, 238.74, 238.75 | D46, D46.0, D46.1, D46.2, D46.20, D46.21, D46.22, D46.4, D46.9, D46.A, D46.B, D46.C, D46.Z |
| Myelofibrosis | 289.83 | D47.4, D75.81 |
| Polycythemia vera | 238.4 | D45 |

ICD-9, ICD-10: International Classification of Diseases, Ninth and Tenth Revision.

**Figure S1. Multi-timepoint complete blood count eligibility and cytopenia criteria.** Time-series plot depicting six scenarios that demonstrate criteria for study eligibility and persistent cytopenia. Time of sequencing is denoted as zero on the x-axis. The blue dashed lines represent one year prior to and after sequencing. The gold dashed line represents 120 days after sequencing. Each circle represents a hemoglobin measurement. To be eligible for the study, participants must have multi-timepoint complete blood count (CBC) data, no laboratory evidence of persistent cytopenia before sequencing, and no diagnosis of myeloid neoplasm before sequencing. Multi-timepoint CBC was defined as at least three CBC measurements, including one within a year of sequencing and two on or after the date of sequencing. The final CBC measurement had to occur at least 120 days after sequencing or the first CBC measurement, whichever came later. CBC measurements occurring greater than one year before sequencing were not included in analysis. Persistent cytopenia was defined as two consecutive observations of a cytopenia in a single lineage at least 120 days apart without an intervening normal measurement. (A) Participant with six eligible and one ineligible CBC measurement who met criteria for multi-timepoint CBC and did not have evidence of an incident, persistent cytopenia. (B) Participant with three eligible CBC measurements who met criteria for multi-timepoint CBC and an incident, persistent cytopenia. (C) Participant with four eligible CBC measurements who met criteria for multi-timepoint CBC and a prevalent, persistent cytopenia. (D) Participant with two eligible CBC measurements who did not meet criteria for multi-timepoint CBC, but did meet criteria for a prevalent, persistent cytopenia. (E) Participant with five eligible CBC measurements who did not meet criteria for multi-timepoint CBC nor persistent cytopenia. (F) Participant with three eligible and one ineligible CBC measurement who did not meet criteria for multi-timepoint CBC, but did meet criteria for a prevalent, persistent cytopenia. The participants labeled (A) and (B) would be included in the case-control study. Participant (C) would be excluded for cytopenia prior to enrollment. Participants (D), (E), and (F) would be excluded for insufficient CBC measurement data.

**Figure S2.** **Flow diagram showing selection of cases and controls across cohorts.** Abbreviations: AML = acute myeloid leukemia; MDS = myelodysplastic syndrome; MF = myelofibrosis; CBC = complete blood count; CHIP = clonal hematopoiesis of indeterminate potential. Participants were screened from (A) All of Us Research Program (N=243,609), (B) Vanderbilt’s BioVU biorepository (N=107,607), and (C) UK Biobank (N=454,033). Participants were excluded for prior AML, MDS or MF diagnoses, insufficient CBC data, or cytopenia at enrollment. Cases and controls were matched 1:3 on age ± 3 years, gender, and any history of smoking within their respective cohort. The same control was able to be matched to multiple cases.


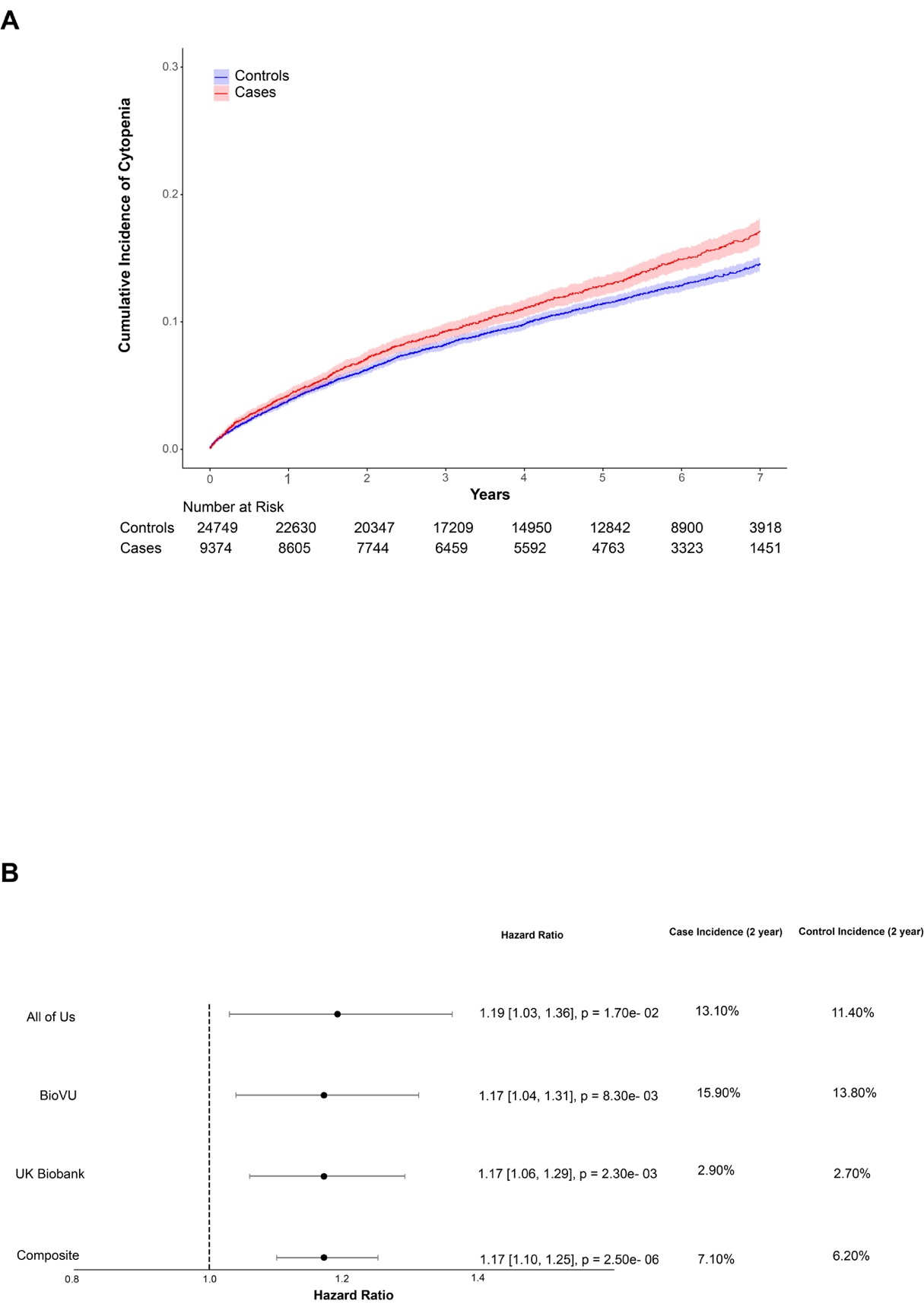


**Figure S3. Risk of incident cytopenia by genotype across cohorts.** Abbreviations: CHIP = clonal hematopoiesis of indeterminate potential. (A) Cumulative incidence of cytopenia in participants with CHIP compared to matched controls across the All of Us Research Program, Vanderbilt’s BioVU biorepository, and UK Biobank. Controls were matched for age ± 3 years, gender and any history of smoking. (B). Risk of incident cytopenia in participants with clonal hematopoiesis of indeterminate potential (CHIP) compared to matched controls across the All of Us Research Program, Vanderbilt’s BioVU biorepository, UK Biobank, and all three cohorts combined with their respective cumulative incidence of cytopenia two years after enrollment. Forest plot indicates HR and 95% confidence intervals.

#

**Figure S4. Risk of incident cytopenia by genotype across cohorts.** Abbreviations: CHIP = clonal hematopoiesis of indeterminate potential. Univariate Cox regression analyses for incident cytopenia by specific CHIP genotypes at time of enrollment for participants with CHIP and controls without CHIP serving as the reference group in (A) All of Us Research Program, (B) Vanderbilt’s BioVU biorepository, and (C) UK Biobank.

**Figure S5.** **Risk of incident cytopenia by baseline characteristic across cohorts.** Abbreviations: VAF = variant allele fraction; CHRS = clonal hematopoiesis risk score; MCV = mean corpuscular volume (femtoliters); RDW = red cell distribution width (%); CHIP = clonal hematopoiesis of indeterminate potential. Univariate Cox regression analyses for incident cytopenia by baseline characteristic at time of enrollment for participants with CHIP without adjustment. The variable high-risk genes (CHRS) indicates a participant had at least one mutation in the following genes: *SRSF2*, *SF3B1*, *ZRSR2*, *IDH1*, *IDH2*, *FLT3*, *RUNX1*, or *JAK2*. The variable high-risk genes indicates a participant had at least one mutation in the following genes: *TP53*, *PPM1D*, *SF3B1*, *SRSF2*, *U2AF1*, *ZRSR2*, *IDH1*, or *IDH2*.

#

**Figure S6. Risk of incident cytopenia stratified by participant risk factor profiles across cohorts.** Cumulative incidence of cytopenia in participants with clonal hematopoiesis of indeterminate potential (CHIP) in (A) All of Us Research Program, (B) Vanderbilt’s BioVU biorepository, and (C) UK Biobank stratified by the number of high-risk features at time of enrollment. High risk features were defined as age ≥ 65 years, male gender, ≥ 2 CHIP mutations, mean corpuscular volume ≥ 100 femtoliters, red cell distribution width ≥ 15%, or the presence of at least one high-risk CHIP mutation (*SRSF2, SF3B1, ZRSR2, IDH1, IDH2, FLT3, RUNX1,* or *JAK2*).


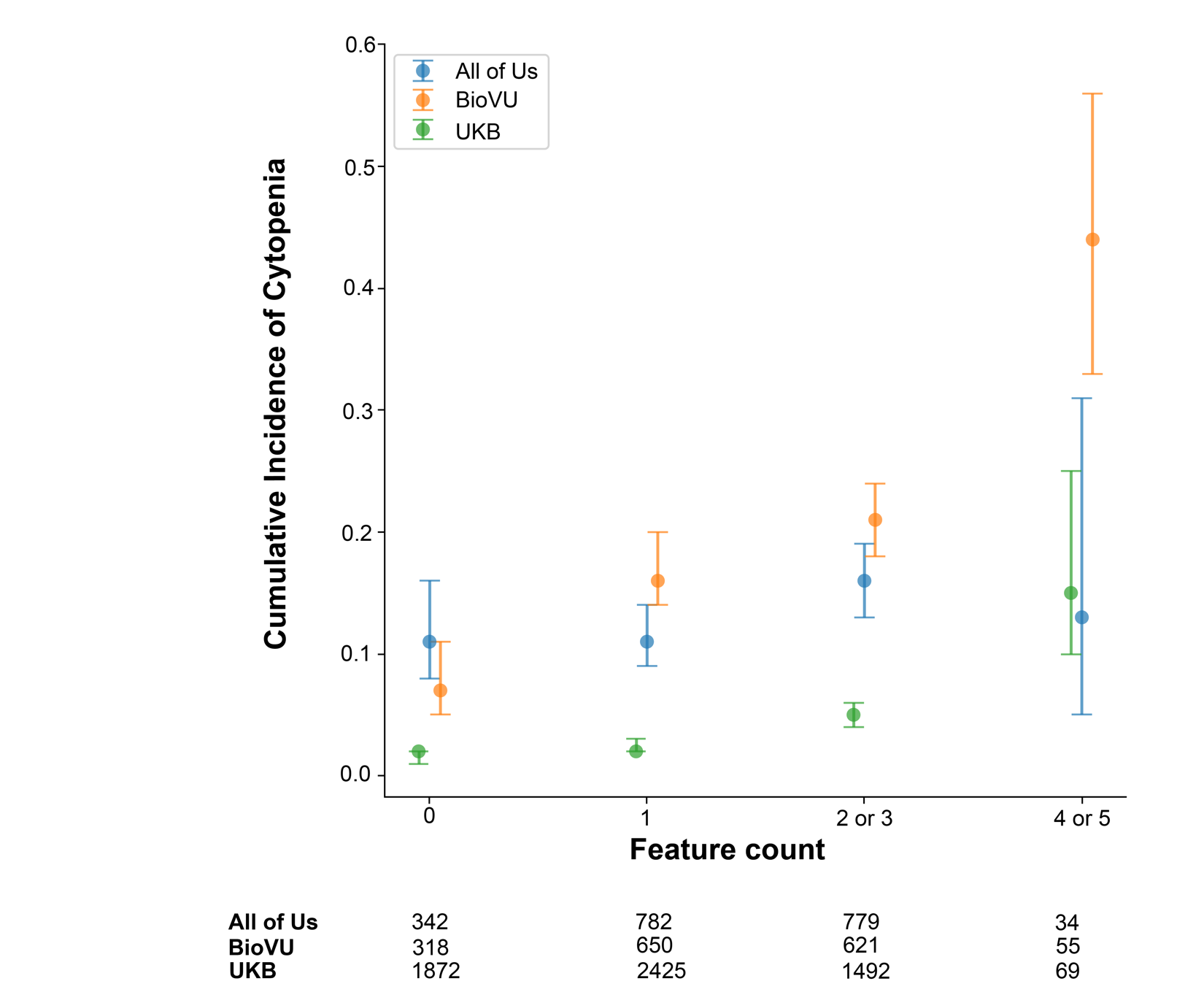


**Figure S7. Cumulative incidence of cytopenia two years after enrollment in participants with CHIP in each biobank stratified by number of high-risk features.**


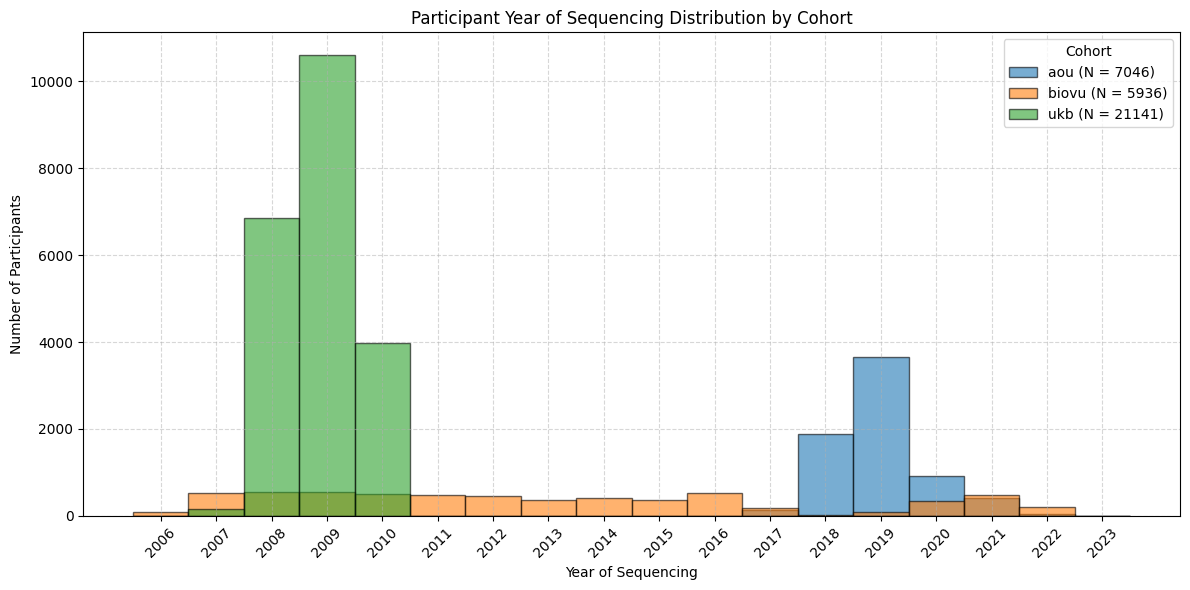


**Figure S8. Participant year of sequencing by cohort of enrollment.** Abbreviations: aou = *All of Us*; biovu = Vanderbilt’s BioVU biorepository; ukb = UK Biobank.

**Figure S9. Risk of incident cytopenia by baseline characteristic in subgroup with at least 5 years of follow-up.** Abbreviations: CHIP = clonal hematopoiesis of indeterminate potential. Univariate Cox regression analyses for incident cytopenia by baseline characteristic at the time of enrollment for participants with CHIP with adjustment for cohort of enrollment. The variable high-risk genes (CHRS) indicates a participant had at least one mutation in the following genes: *SRSF2*, *SF3B1*, *ZRSR2*, *IDH1*, *IDH2*, *FLT3*, *RUNX1*, or *JAK2*. The variable high-risk genes* indicates a participant had at least one mutation in the following genes: *TP53*, *PPM1D*, *SF3B1*, *SRSF2*, *U2AF1*, *ZRSR2*, *IDH1*, or *IDH2*.

**Figure S10. Risk of incident cytopenia by genotype in subgroup with at least 5 years of follow-up.** Univariate Cox regression analyses for incident cytopenia by specific CHIP genotypes at the time of enrollment for participants with CHIP and controls without CHIP serving as the reference group. Analyses were adjusted for age and cohort of enrollment.

**A**

**B**

**
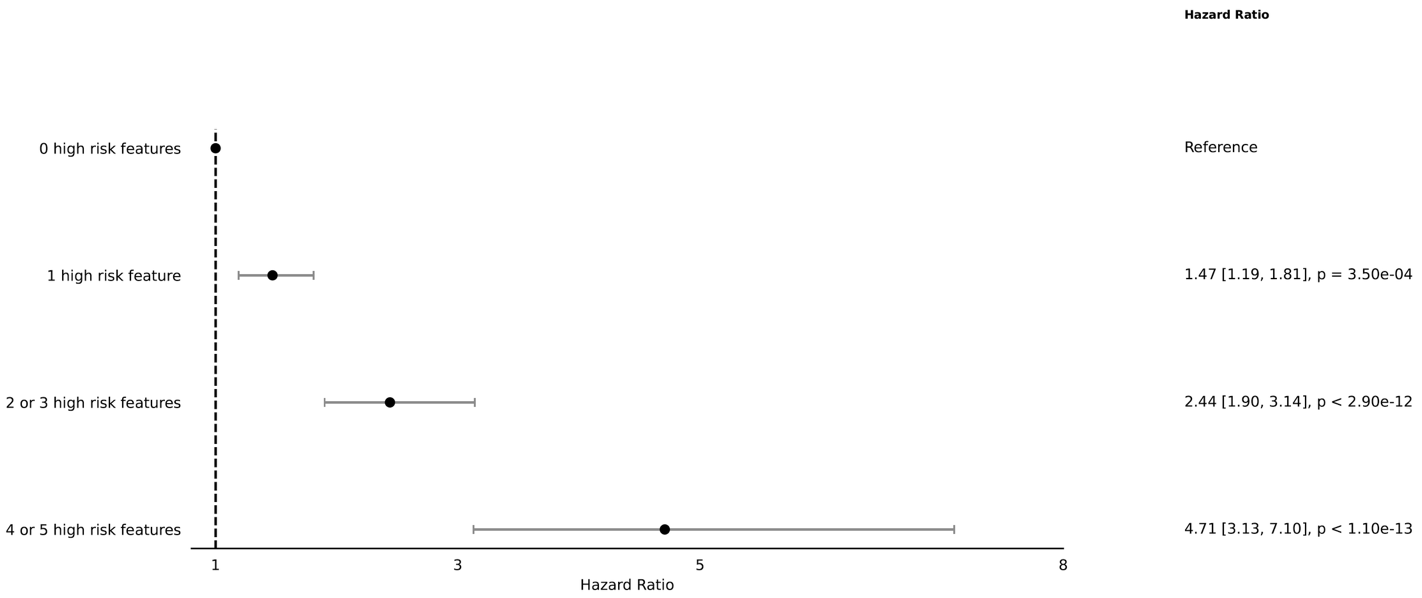
**

**Figure S11. Risk of incident cytopenia stratified by participant risk factor profiles in subgroup with at least 5 years of follow-up.** (A) Cumulative incidence curve for cytopenia in participants with CHIP across All of Us Research Program, Vanderbilt’s BioVU biorepository, and UK Biobank stratified by the number of high-risk features they had at time of enrollment. High risk features were defined as age ≥ 65 years, male gender, ≥ 2 CHIP mutations, MCV ≥ 100 femtoliters, RDW≥15%, or the presence of at least one high-risk CHIP mutation (*SRSF2*, *SF3B1*, *ZRSR2*, *IDH1*, *IDH2*, *FLT3*, *RUNX1*, or *JAK2*). (B) Hazard ratios for incident cytopenia were calculated for high-risk feature strata using Cox proportional hazards models adjusted for cohort, age, sex, gender, and smoking history. Hazard ratios were calculated in a model with zero high-risk features as the reference population. Forest plot indicates HR and 95% confidence intervals.

**A**

**B**


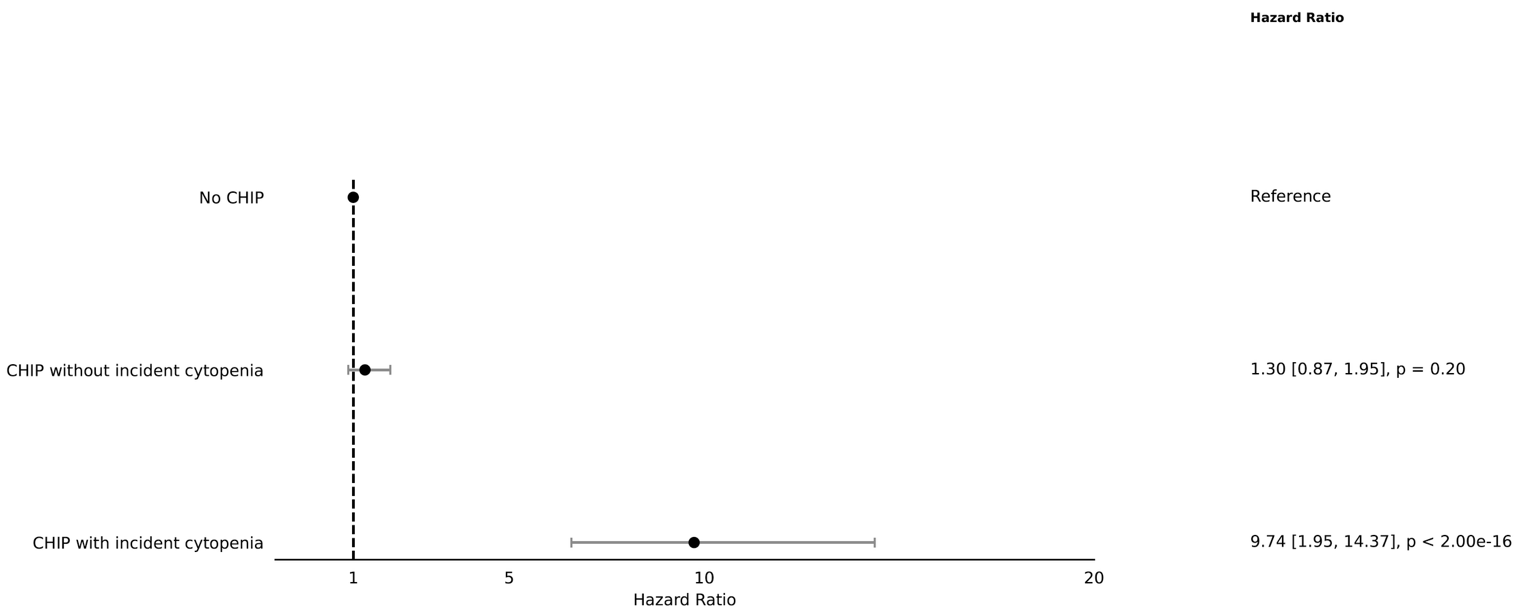


**Figure S12. Risk of incident AML, MDS, MF stratified by CHIP status at enrollment, incident cytopenia.** Risk of incident AML, MDS, MF stratified by CHIP status at enrollment, incident cytopenia, and mutation. Abbreviations: AML = acute myeloid leukemia; MDS = myelodysplastic syndrome; MF = myelofibrosis; MN = myeloid neoplasm; CHIP = clonal hematopoiesis of indeterminate potential. (A) Risk of incident AML, MDS, MF in participants with CHIP stratified by incident cytopenia status compared to matched controls across All of Us Research Program, Vanderbilt’s BioVU biorepository, and UK Biobank. (B) Hazard ratios for incident AML, MDS, MF were calculated for cases and controls stratified by incident cytopenia using Cox proportional-hazards models adjusted for cohort, age, sex, gender, and smoking history. Hazard ratios were calculated in a model with participants without CHIP as the reference population. Forest plot indicates HR and 95% confidence intervals.
